# Autism spectrum disorder associated with low serotonin in CSF and mutations in the *SLC29A4* plasma membrane monoamine transporter (PMAT) gene

**DOI:** 10.1186/2040-2392-5-43

**Published:** 2014-08-13

**Authors:** Dea Adamsen, Vincent Ramaekers, Horace TB Ho, Corinne Britschgi, Véronique Rüfenacht, David Meili, Elise Bobrowski, Paule Philippe, Caroline Nava, Lionel Van Maldergem, Rémy Bruggmann, Susanne Walitza, Joanne Wang, Edna Grünblatt, Beat Thöny

**Affiliations:** 1 Division of Metabolism, Department of Pediatrics, University of Zürich, Zürich 8032, Switzerland; 2 Affiliated with the Neuroscience Center Zürich, University of Zürich and ETH Zürich (ZNZ), Zürich 8000, and the Children’s Research Center (CRC), Zürich 8032, Switzerland; 3 Centre of Autism Liège and Division of Pediatric Neurology, University Hospital Liège, Liège 4000, Belgium; 4 Department of Pharmaceutics, University of Washington, Seattle 98195, WA, USA; 5 Division of Clinical Chemistry and Biochemistry, Department of Pediatrics, University of Zürich Zürich 8032, Switzerland; 6 University Clinics of Child and Adolescent Psychiatry, University of Zürich, Zürich 8050, Switzerland; 7 Department of Genetics, Cytogenetics and human Genetics, Pitié-Salpêtrière Hospital, Paris 75651, France; 8 Centre for Human Genetics, University of Franche-Comté, Besançon 25030, France; 9 Functional Genomics Center Zürich, ETH Zürich/University of Zürich, Zürich 8057, Switzerland; 10 current address: Interfaculty Bioinformatics Unit, University of Bern/Swiss Institute of Bioinformatics, Bern 3012, Switzerland; 11 Affiliated with the Neuroscience Center Zürich, University of Zürich and ETH Zürich (ZNZ), Zürich 8000, Switzerland; 12 Affiliated with the Zürich Center for Integrative Human Physiology (ZIHP), University of Zürich, Zürich 8000, Switzerland

**Keywords:** Autism spectrum disorder, Serotonin end-metabolite 5-hydroxyindolacetic acid, SERT, PMAT

## Abstract

**Background:**

Patients with autism spectrum disorder (ASD) may have low brain serotonin concentrations as reflected by the serotonin end-metabolite 5-hydroxyindolacetic acid (5HIAA) in cerebrospinal fluid (CSF).

**Methods:**

We sequenced the candidate genes S*LC6A4* (SERT), *SLC29A4* (PMAT), and *GCHFR* (GFRP), followed by whole exome analysis.

**Results:**

The known heterozygous p.Gly56Ala mutation in the *SLC6A4* gene was equally found in the ASD and control populations. Using a genetic candidate gene approach, we identified, in 8 patients of a cohort of 248 with ASD, a high prevalence (3.2%) of three novel heterozygous non-synonymous mutations within the *SLC29A4* plasma membrane monoamine transporter (PMAT) gene, c.86A > G (p.Asp29Gly) in two patients, c.412G > A (p.Ala138Thr) in five patients, and c.978 T > G (p.Asp326Glu) in one patient. Genome analysis of unaffected parents confirmed that these PMAT mutations were not *de novo* but inherited mutations. Upon analyzing over 15,000 normal control chromosomes, only *SLC29A4* c.86A > G was found in 23 alleles (0.14%), while neither c.412G > A (<0.007%) nor c.978 T > G (<0.007%) were observed in all chromosomes analyzed, emphasizing the rareness of the three alterations. Expression of mutations PMAT-p.Ala138Thr and p.Asp326Glu *in cellulae* revealed significant reduced transport uptake activity towards a variety of substrates including serotonin, dopamine, and 1-methyl-4-phenylpyridinium (MPP^+^), while mutation p.Asp29Gly had reduced transport activity only towards MPP^+^. At least two ASD subjects with either the PMAT-Ala138Thr or the PMAT-Asp326Glu mutation with altered serotonin transport activity had, besides low 5HIAA in CSF, elevated serotonin levels in blood and platelets. Moreover, whole exome sequencing revealed additional alterations in these two ASD patients in mainly serotonin-homeostasis genes compared to their non-affected family members.

**Conclusions:**

Our findings link mutations in *SLC29A4* to the ASD population although not invariably to low brain serotonin. PMAT dysfunction is speculated to raise serotonin prenatally, exerting a negative feedback inhibition through serotonin receptors on development of serotonin networks and local serotonin synthesis. Exome sequencing of serotonin homeostasis genes in two families illustrated more insight in aberrant serotonin signaling in ASD.

## Background

Autism spectrum disorder (ASD), which includes infantile autism (Kanner syndrome), Asperger’s syndrome, and pervasive developmental disorder – not otherwise specified (PDD-NOS), are a group of complex and lifelong neuro-developmental disorders characterized by deficits within three core domains, social interaction, verbal and non-verbal communication, as well as limited interests and repetitive, stereotypic behavior [1,2]. Numerous family and twin studies have reported that ASD are highly heritable disorders and suggest that approximately 90% of variance is attributable to genetic factors [3], while other studies reported a much more moderate genetic heritability and a substantial shared twin environmental component [4]. Despite the strong genetic evidence, the identity and number of genes involved or responsible for ASD are not yet known [1]. Nevertheless, it is generally assumed that the genes involved in the pathogenesis of autism are implicated in the overall processes of neuronal signal transmission, including molecules such as synaptic scaffolding proteins, cell adhesion molecules, protein involved in second-messenger systems, secreted proteins, receptors, and transporters [5].

Serotonin, as a monoamine neurotransmitter and hormone, plays numerous roles and is a critical modulator of neuronal interaction that supports diverse behaviors and physiological processes, and acts via different specific transporters, receptors, and intracellular signaling pathways. Multiple lines of evidence implicate abnormal serotonergic signaling in psychiatric and neuro-developmental pathogenesis. However, the entire serotonin system is poorly defined and is far from a complete understanding. Laboratory analyses of cerebrospinal fluid (CSF) describe a frequency of up to 20% of patients with altered serotonin end-metabolite 5-hydroxyindolacetic acid (5HIAA) in neurological disorders, including subjects with ASD [6] (for a broader overview on monoamine neurotransmitter disorders see reference [7] and references therein). Serotonin re-uptake transporter genes, such as SERT-*SLC6A4* and PMAT-*SLC29A4*, are of special interest for ASD for at least three reasons [8]: i) Serotonin has been shown to regulate the development of the central nervous system [9] and to be involved in a broad spectrum of behavioral and psychological processes (e.g., social behavior, aggression and anxiety) and various psychiatric disorders [10]. ii) Several studies have reported that approximately one-third of all autistic patients have elevated levels of whole blood (or platelet) serotonin [11,12]. Hyperserotonemia, which is believed to be caused by abnormal maturation of the serotonin system [9,12], is also suggested to be either directly or indirectly responsible for the immune abnormalities observed among autistic subjects [13]. Additionally, developmental changes in brain serotonin synthesis capacity measured by PET using the radioactive marked serotonin precursor (α[^11^C]-methyl-L-tryptophan) showed a diminished capacity of whole-brain or regional brain serotonin synthesis in autistic children compared to non-autistic children, thus suggesting that developmental regulation of serotonin synthesis is involved in the pathogenesis of autism [14]. iii) Increased repetitive behaviors and irritability were observed in autistic patients when subjected to a dietary depletion of the serotonin precursor tryptophan due to an expected reduction in extracellular serotonin availability [15]. Further support for a link between brain hyposerotonemia and a possible relevance to autism comes from mice deficient in neuronal tryptophan hydroxylase 2 (*Tph2*^*-/-*^), which lack brain serotonin, and showed substantial deficits in social interaction and communication, and also displayed highly repetitive and compulsive behaviors [16]. Increased extracellular serotonin availability due to administration of selective serotonin re-uptake inhibitors led to reduced symptoms of irritability and rigid-compulsive behavior in individuals with ASD [15,17]. Altogether, these findings emphasize that abnormal serotonergic transmission may be important in the pathogenesis of autism [18,19]. Due to the serotonin hypothesis of autism, genes encoding proteins involved in brain serotonin metabolism and neurotransmission have received more attention than other categories of genes [20].

In this context, we found that isolated low brain serotonin concentration, as reflected by the 5HIAA in the CSF, is associated with PDD-NOS and the functional (heterozygous) c.167G > C (p.G56A) mutation of the serotonin re-uptake transporter gene (SERT/*SCL6A4*) combined with a homozygous long (L/L) SERT gene-linked polymorphic promoter (5-HTTLPR) region [21]. Moreover, daily treatment with serotonin precursor 5-hydroxytryptophan and aromatic amino acid decarboxylase (AADC) inhibitor carbidopa led to clinical improvements and normalization of the 5HIAA levels in the CSF and urine, indicating that the brain serotonin turnover was normalized [22]. In an attempt to gain some insight into the brain serotonin physiology and underlying mechanisms of abnormal brain metabolism, we report in patients with ASD and low brain 5HIAA mutations in the serotonin transporter *SCL29A4*, an observation that may provide some bases for improving the application of various therapeutic tools.

## Methods

### Patients involved and work-flow

**Figure 1:**
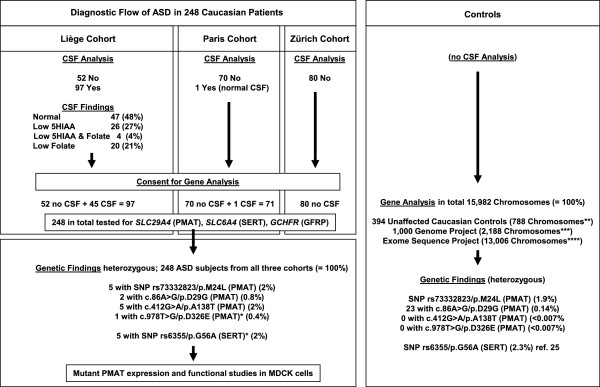
Diagnostic flow-chart of ASD in 248 patients (see text for details). *One patient with p.D326E (PMAT) and p.G56A (SERT). **Using genomic DNA from an independent cohort from the Children’s Hospital in Zurich of 394 unaffected control subjects (a total of 788 chromosomes). ***see http://www.1000genomes.org; phase1 integrated release version3 20120430. ****see http://evs.gs.washington.edu/EVS/; accessed August 2012 (EVS-v.0.0.14); see also reference [23].

Figure 1 depicts a flow-chart of the study, including selection of the participating patients from the three cohorts in Liège, Paris, and Zürich, the results of CSF findings in 98 patients, and the collection of DNA samples from patients and controls. All patients have been diagnosed with ASD according to DSM-IV and ICD-10 criteria followed by comprehensive investigations using the Autism Diagnostic Interview and Autism Diagnostic Observation Schedule test procedure. Further details can be obtained from Additional file 1.

#### Screening for mutations in the human SLC29A4 gene

Intronic primers were designed with the online program ExonPrimer (http://ihg.gsf.de/ihg/ExonPrimer.html) to flank the exons in the human *SLC29A4* gene (PMAT/*SLC29A4*; OMIM: 609149; ENST00000297195) (for more details see Additional file 2: Table S1). PCR reactions were performed in a final volume of 25 μL containing 1 μL of genomic DNA template (100 ng), 1 μL of each primer (forward and reverse) (5 μM), 0.5 μL 10 mM deoxyribonucleoside triphosphates, 0.2 μL Hot FirePolHotstart DNA polymerase (Solis Biodyne, Tartu, Estonia), 1.5 μL 25 mM MgCl_2_, 2.5 μL 10× reaction buffer (Mg^2+^ free from Solis Biodyne), and 5 μL 5× Q-solution (Qiagen, Hilden, Germany). The PCR reactions were performed in a GeneAmp PCR System 9700 from (Life Technologies, Zug, Switzerland) using standard thermal cycling: hot start activation at 95°C for 15 minutes, followed by 35 cycles of denaturation 95°C for 1 minute, annealing 55°C for 45 seconds, and extension at 72°C for 1 minute, a final extension was performed at 72°C for 10 minutes and termination at 4°C. The same forward and reverse primers as mentioned above were used to sequence the amplified PCR products with BigDye Terminator v1.1 Cycle Sequencing Kit (Applied Biosystems). Sequencing PCR was performed in a 10 μLreaction mixture consisting of 0.5 μL PCR product, 0.8 μL primer (5 μM), 1.5 μLBigDye, and 1.25 μL5× sequencing buffer (BigDye and 5 × sequencing buffer were both from BigDye Terminator v1.1 Cycle Sequencing Kit from Applied Biosystems) using standard thermal cycling: (activation at 95°C for 1 min, followed by 25 cycles of 96°C for 10 seconds, 50°C for 5 seconds, 60°C for 3 minutes and termination at 4°C). After end run, 15 μL H_2_O was added to the sequencing reactions before purified by gel filtration (using MultiScreen HV 96-well filter plates (Merck Millipore, Darmstadt, Germany) with Sephadex G-50 (GE Healthcare, Little Chalfont, UK) and analyzed on a 3130*xl* Genetic Analyzer (Applied Biosystems). Sequencing results were compared with wild-type sequence of the *SLC29A4* gene by using Mutation Surveyor (Demo) Software v3.20 from SoftGenetics (State College, PA, USA; Transcript ID: ENST00000297195 or accession number NM_001040661). In addition, we have compared all the polymorphisms with the data of the 1000 Genomes Project (http://www.1000genomes.org; phase1 integrated release version3 20120430) and the Exome Variant Server of the Exome Sequencing Project (http://evs.gs.washington.edu/EVS/; accessed August 2012; see also reference [23]).

#### Screening for mutations in the human SLC6A4 gene

Screening of genomic DNA for mutations as well as detection of non-coding VNTR gene polymorphisms (5-HTTLPR and STin2 VNTR) in the human *SLC6A4* gene (SERT/*SLC6A4*; OMIN: 182138; ENST00000394821) were performed as described previously [21].

#### Generation of mutant PMAT by site-directed mutagenesis

To facilitate the determination of membrane localization, PMAT mutants were constructed using yellow fluorescence protein (YFP)-tagged wild-type human PMAT-cDNA as template. YFP was tagged at the N-termini of the wild-type and mutant PMAT transporters, as previous studies have shown that the YFP tagging had no effect on substrate selectivity and kinetic behaviors of the transporter [24]. The wild-type human PMAT-cDNA was previously sub-cloned into the YFP vector pEYFP-C1 (Clontech, Palo Alto, CA, USA) [25]. PMAT-D29G, A138T, and D326E mutations were introduced into wild-type PMAT/pEYFP-C1 vector by site-directed mutagenesis and verified by DNA sequencing.

#### Stable expression in Madin-Darby canine kidney (MDCK) cells

YFP-tagged mutant constructs were transfected into MDCK cells using Lipofectamine 2000 transfection reagent (Invitrogen, Carlsbad, CA, USA). Stably transfected cell lines were obtained by culturing cells in minimal essential medium containing 10% fetal bovine serum and G418 (1,000 μg/mL). Empty pEYFP-C1 vector was transfected into MDCK cells to obtain the control cell line. After 2 to 3 weeks of drug selection, fluorescence-positive cells were purified by a FACS Vantage SE sorter (BD Biosciences, San Jose, CA, USA) at the Cell Analysis Center at the University of Washington, Health Sciences Center. The sorted cells were cultured and maintained in minimal essential medium containing G418 (200 μg/mL).

#### Confocal fluorescence microscopy

To determine the cellular localization of YFP-tagged mutant transporters, ~2 × 10^5^ cells were grown on top of a microscope cover glass in 6-well plates (Falcon, BD Biosciences, Bedford, MA, USA) for 2 to 3 days until confluent. Cells were mounted onto microscope glass slides with Fluoromount-G (Electron Microscopy Sciences, Hatfield, PA, USA) and visualized with a Leica SP1 confocal microscope equipped with an argon laser as the light source at the Keck Microscopy Facility at the University of Washington. Images were captured by excitation at 488 nm and emission at 515 nm.

#### Functional characterization in MDCK cells

Stably transfected MDCK cells were plated in 24-well plates and allowed to grow for 2 to 3 days until confluent. Growth medium was aspirated and each well was rinsed once with Krebs-Ringer-Henseleit buffer (5.6 mM glucose, 125 mM NaCl, 4.8mMKCl, 1.2 mM KH_2_PO_4_, 1.2 mM CaCl_2_, 1.2 mM MgSO_4_, 25 mM HEPES, pH 7.4) and preincubated in the same buffer for 15 min at 37°C. Transport assays were performed at 37°C by incubating cells in KRH buffer containing a ^3^H-labeled ligand. [^3^H]MPP^+^ (85 Ci/mmol) was obtained from American Radiolabeled Chemicals, Inc. (St. Louis, MO, USA). [^3^H]5-HT (5-hydroxy-[1,2-^3^H] tryptamine creatinine sulfate, 28.1 Ci/mmol) and [^3^H] dopamine (3,4-dihydroxy-[2,5,6-^3^H] phenylethylamine, 51.3 Ci/mmol) were obtained from PerkinElmer (Life Sciences Inc., Waltham, MA, USA). All other chemicals were obtained from Sigma (St. Louis, MO, USA). Uptake was terminated by washing the cells three times with ice-cold KRH buffer. Cells were then solubilized with 0.5 mL of 1 N NaOH and neutralized with 0.5 mL of 1 N HCl. Radioactivity in the cell lysate was quantified by liquid scintillation counting. Protein concentration in each well was measured using BCA protein assay kit (Pierce, Rockford, IL, USA) and the uptake in each well was normalized to its total protein content. In all studies, cells transfected with an empty vector served as a background control. Transporter-specific uptake was calculated by subtracting the background uptake in vector-transfected cells. For all uptake experiments, data were expressed as the mean ± SD from three independent experiments (n = 3) with different cell passages. For each experiment, uptake was carried out in triplicates on the same plate. Where applicable, *P* values were obtained through Student’s *t*-test using Prism software (GraphPad Software Inc., La Jolla, CA, USA). *P* values <0.05 were considered significant.

#### Isolation of plasma membrane proteins by cell surface biotinylation

Stably transfected MDCK cells were plated onto 60 mm plates and cultured until confluent. Cells were washed twice with 3 mL of ice-cold PBS/CM (138 mM NaCl, 2.7 mMKCl, 8 mM Na_2_HPO_4_, 1.5 mM KH_2_PO_4_, 0.1 mM CaCl_2_, 1 mM MgCl_2_, pH 8.0). Biotinylation was carried out on ice by incubation with 1 mL of ice-cold PBS/CM containing a membrane-impermeable biotinylation reagent Sulfo-NHS-SS-biotin (0.5 mg/mL) (Pierce). After two successive 20 min incubations at 4°C with freshly prepared NHS-SS-biotin and gentle shaking, cells were briefly rinsed with 3 mL of PBS/CM containing 100 mM glycine. Cells were further incubated at 4°C with the same solution for 20 min to ensure complete quenching of the unreacted NHS-SS-biotin. Cells were then solubilized on ice by incubating in 1 mL of lysis buffer containing 20 mM Tris, 150 mMNaCl, 1 mM EDTA, 1% Triton X-100, 1 mMphenylmethyl-sulfonyl fluoride, and Protease Inhibitors Cocktail (Roche) for 1 h with occasional vortexing. Protein concentrations were measured from the supernatant lysate and 50 μL of UltraLink Immobilized NeutrAvidin protein (Pierce) was then added to the supernatant for the isolation of membrane proteins. Membrane proteins were subjected to Western blot using a mouse monoclonal anti-yellow fluorescent protein antibody (JL-8) (BD Biosciences) at 1:1,000 dilution, followed by horseradish peroxidase-conjugated goat anti-mouse IgG (1:20,000 dilution). The chemiluminescent signals in the Western blots were detected by using SuperSignal West Pico Chemiluminescent Substrate (Pierce) followed by exposure of the blots to X-ray films. Band intensity was quantified by densitometry using the ImageQuant software (Molecular Dynamics, Thermo Fisher Scientific Inc, Rockford, IL, USA). As reported previously [24], double or multiple protein bands around the expected molecular size (~75 kDa) were observed for the YFP-tagged PMAT proteins, which could be due to differential glycosylation of PMAT.

#### Whole exome sequencing and sequence analysis

Whole exome sequencing was performed in the two ASD cases MT and PL with isolated low serotonin and carrying PMAT-A138T or D326E, plus their unaffected parents and siblings (for more details see Additional file 3: Figure S4). The color space sequence reads (paired end 50 × 35) were mapped to the human genome assembly hg19 (http://hgdownload.cse.ucsc.edu/goldenPath/hg19/bigZips/chromFa.tar.gz) using the software Bioscope 1.2.1 from Applied Biosystems (Life Technologies, Carlsbad, CA, USA). After mapping, the single nucleotide polymorphisms (SNPs) were called by using the DiBayes algorithm (Life Technologies) with high and medium stringency settings. The obtained SNPs were classified into intronic, intergenic, coding (synonymous and non-synonymous amino acid substitutions), and splice-site variants with a custom software pipeline. Furthermore, all SNPs were compared to dbSNP build 132, OMIM, and clinically relevant mutation.

## Results

### Analysis of CSF and sequencing of *SLC6A4*, *SLC29A4*, and *GCHFR* genes

To follow-up our previous findings or association [21], i.e., ASD and abnormally low brain serotonin metabolites with potential treatment possibilities, we analyzed a Caucasian cohort of 248 patients diagnosed with ASD – a diagnostic flow-chart of these patients is depicted in Figure 1. We sequenced genomic DNA using a genetic candidate gene approach including genes encoding SERT (*SCL6A4*), plasma membrane monoamine transporter PMAT (*SLC29A4*), and GTP cyclohydrolase I feedback regulatory protein GFRP (*GCHFR*), as they might be involved in serotonin homeostasis. The latter was proposed to be involved specifically in serotonin neurons and not within dopamine neurons to regulate the tetrahydrobiopterin biosynthesis [26]. Up to now, no mutations have been reported within the *GCHFR* and *SLC29A4* genes; however, *SLC29A4* is located on chromosome position 7p22.1 [25], where association studies identified a susceptibility locus for ASD [27]. Among the initially diagnosed 300 ASD-affected patients, a subset of 97 subjects (=100%) underwent a lumbar puncture for CSF analysis of monoamine metabolites, pterins, and folate levels after obtaining informed consent (Additional file 1). The CSF analysis indicated normal values in 47 (48%) subjects and isolated low 5HIAA concentration in 26 (27%). Further, isolated low folate in CSF was found in 20 subjects (21%), and both low folate and low 5HIAA values in CSF were present in 4 subjects (4%). Pterin levels among all patients were normal. Mutations in genes encoding AADC and monoamine oxidase A were considered unlikely based on normal catecholamines, homovanillic acid, 5OH-tryptophan, and 5HIAA levels in CSF for the subjects analyzed, and were therefore discarded.

Among the genetically investigated 248 ASD cases, all exons and exon-intron boundaries of *SLC6A4*, *SLC29A4*, and *GCHFR* were sequenced using standard methods (Additional file 1). Further, no mutations in *GCHFR* were found in the study cohort (not shown). However, we identified a single heterozygous non-synonymous mutation in the *SLC29A4* gene in eight ASD patients (3.2% in our cohort of 248 subjects): two carrying c.86A > G, five patients, including two identical twin brothers, with c.412G > A, and one that had the c.978 T > G sequence alteration, determining p.Asp29Gly, p.Ala138Thr, and p.Asp326Glu residue changes of PMAT, respectively (OMIM 609149; ENST00000297195) (Additional file 4: Figure S1). Although the identical twins and three other patients with the most frequent occurring PMAT p.Ala138Thr mutation came from different unrelated families, we have not performed an analysis of genetic markers to confirm a founder effect. In addition, five subjects (2%) of the 248 ASD-affected patients were heterozygous for the *SLC6A4* base change c.167G > C, encoding p.Gly56Ala (Additional file 2: Table S2), which did not differ significantly from the frequency of 2.3% in the control populations, and has already been associated with autism and rigid-compulsive behaviors [28]. In a single patient (PL), a double heterozygosity was observed, since both *SLC6A4* c.167G > C and *SLC29A4* c.978 T > G were found (Additional file 2: Table S2). Whereas the *SLC6A4* c.167G > C (SERT-G56A) alteration is a known mutation with a gain of function regarding serotonin re-uptake [28], mutations within the *SLC29A4* gene have not been described before, thus making these three novel mutations the first of their kind. Using genomic DNA from an independent group of 394 unaffected control subjects (a total of 788 chromosomes) (Additional file 1), we ruled out the possibility that the *SLC29A4* mutations c.86A > G, c.412G > A, and c.978 T > G are frequent SNPs, as none of these variants were detected in our control cohort. Additionally, after a search in the 1000 Genomes Project and Exome Sequence Project data (a total of 2,188 plus 13,006 chromosomes respectively) only *SLC29A4* c.86A > G was found in 23 alleles (<0.16%) in the database of the Exome Variant Server, while neither c.412G > A (<0.007%) nor c.978 T > G (<0.007%) were observed in all chromosomes analyzed (Additional file 1). These results emphasized the rareness of the three mutations described here. In addition to *SLC29A4* c.86A > G, c.412G > A, and c.978 T > G mutations, PMAT-M24L (rs73332823), which was found in 2% of our ASD-affected cohort (5/248) but also in 1.4% of our control chromosomes (43/2976; not shown), strongly suggested that it simply represents a common SNP.

### Evolutionary conservation analysis and functional evaluation of PMAT mutants

**Figure 2:**
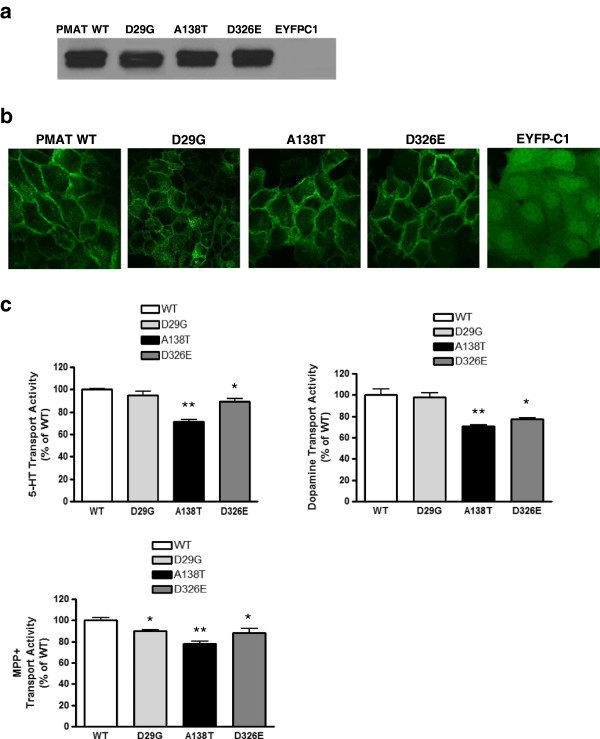
**Discovery and functional characterization of PMAT mutations from ASD patients.** Out of 248 patients diagnosed with ASD and normal or isolated low 5HIAA in the CSF, 8 patients were carrying a heterozygous mutation in the *SLC29A4* gene encoding for PMAT: c.86A > G/p.D29G in 2 subjects, c.412G > A/p.A138T in 5 subjects, and c.978 T > G/p.D326E in 1 subject, indicating a cumulative prevalence of 3.2% in our analyzed cohort of ASD patients (Additional file 4: Figure S1). **(a)** Cell surface biotinylation and Western blot analysis demonstrated comparable plasma membrane expression of PMAT-wildtype (wt), PMAT-D29G, PMAT-A138T, and PMAT-D326E proteins. Empty pEYFP-C1 vector was transfected into MDCK cells to generate the control cell line. **(b)** Confocal microscopy imaging of MDCK cells expressing PMAT-wt, PMAT-D29G, PMAT-A138T, PMAT-D326E, and empty pEYFP-C1 vector. The three mutated PMAT proteins revealed normal localization in the plasma membrane similar to PMAT-wt. Scale bars: 20 μm. **(c)** Serotonin (5-HT), dopamine and 1-methyl-4-phenylpyridinium (MPP^+^) transport activity were significantly reduced in MDCK cells transfected with PMAT-A138T and PMAT-D326E. For all the transport activity experiments, the values are indicated in mean ± SD from three independent experiments (n = 3) with different cell passages. For each experiment, uptake was carried out in triplicates on the same plate. Significant difference from the corresponding value for PMAT-wt (100% transport activity) is indicated by asterisks: *, *P* < 0.05 and **, *P* < 0.01 (Student’s *t*-test).

The newly discovered amino acid changes p.D29G, p.A138T, and p.D326E in PMAT are located in the N-terminus, third membrane-spanning domain and the large cytoplasmic loop between transmembrane domains 6 and 7 in the PMAT protein respectively (Additional file 5: Figure S2). Alignment of the fully annotated PMAT protein sequences from seven species showed that D29 and D326 are well conserved across mammalian species whereas A138 is less conserved (Additional file 6: Figure S3). Using two publically available prediction algorithms, MutationTaster [29] and PolyPhen2 [30], the D29G and the D326E mutations were predicted to have more deleterious effects than the A138T mutation (Additional file 2: Table S3). These regions have previously been shown to contain important sites for PMAT substrate recognition and transport [24,31]. Nevertheless, to experimentally investigate the functional impact of these mutations, the PMAT alterations p.D29G, p.A138T, and p.D326E were constructed, expressed in MDCK cells and analyzed in terms of subcellular localization and transport activity in comparison with PMAT-wild-type (Figure 2a–c). Cell surface biotinylation and Western blot analysis demonstrated comparable plasma membrane expression of PMAT-wildtype and PMAT-D29G, PMAT-A138T, and PMAT-D326E mutant proteins (Figure 2a). When expressed in MDCK cells and observed by confocal microscopy, the three mutated PMAT proteins were located in the plasma membrane similar to PMAT-wildtype (Figure 2b), demonstrating that these single amino acid alterations p.D29G, p.A138T, and p.D326E do not alter protein stability and trafficking to the plasma membrane. To assess the functional consequence of the PMAT mutations, we compared the uptake activity of wild type and mutant proteins towards a variety of radiolabeled substrates including serotonin, dopamine, and MPP^+^ (1-methyl-4-phenylpyridinium) (Figure 2c). The functional studies revealed that both PMAT-A138T and PMAT-D326E exhibited similarly reduced transport activity towards all three substrates tested (serotonin, dopamine, and MPP^+^), suggesting that the two mutants induce an overall reduction in transport activity and are functionally indistinguishable from each other. In contrast, PMAT-D29G showed serotonin and dopamine transport activities similar to that of PMAT-wildtype, but its transport activity toward MPP^+^ was found to be reduced similar to that of PMAT-A138T and PMAT-D326E. The more-than-predicted damaging effect of the A138T mutation is likely due to its position on TM 3 (Additional file 5: Figure S2) which lies within a region that has previously been shown to contain important sites for PMAT substrate recognition and transport [24,31]. In addition, previous analysis of natural variations in a number of human membrane transporter genes has suggested that TM regions have special evolutionary and functional constraints. Compared to the loop regions, there are much less variations observed in the TM regions, even in evolutionarily unconserved residues [32].

### Whole exome sequencing of families with detected PMAT mutations

While SERT-G56A is known to cause an up-regulation for serotonin transport (gain of function) [28], our functional data suggest that the two PMAT mutations (PMAT-A138T and PMAT-D326E) induce reduced transport activity towards serotonin. All sequence alterations we have identified in *SLC6A4* and *SLC29A4* were present in their heterozygous states (Additional file 4: Figure S1). From segregation studies by genomic DNA sequencing analysis of the parents of the ASD-affected patients, it appears that the mutations in *SLC6A4* and *SLC29A4* are inherited and are not *de novo* mutations*.* Like in several locus-specific CNV-related ASD, heritability does not seem to follow a simple autosomal dominant mode of inheritance and suggests rather a complex heritability and haplotype association. Furthermore, we observed elevated serotonin levels both in blood (ng/mL) and platelets (ng/×10^9^) in two of the ASD-affected patients MT and PL (Table 1), which also were found to carry common non-coding variable number tandem repeat (VNTR) polymorphisms within the *SLC6A4* gene (5-HTTLPR L/S and STin2 VNTR 10/12 variants; Additional file 2: Table S2 and Additional file 3: Figure S4). The elevated serotonin found in blood and platelets supports previous studies showing that one-third of all ASD-affected individuals have hyperserotonemia [11,12].

**Table 1:** Summary of metabolic findings in patient MT (Asperger’s) and PL (PDD-NOS)

**Patient**	**Age (years)**	**5HIAA (nmol/L)**	**HVA (nmol/L)**	**HVA/5HIAA (ratio)**	**Serotonin**	
					**Blood (ng/mL)**	**Platelets (ng/×10**^**9**^**)**
MT	9.6	64.7	389.6	6.0	287	1041
PL	6.2	73.6	399.8	5.4	221	675
Ref*	5–10*	133 (88–178)*	523 (144–801)*	1.5–3.5*	50–220**	125–500**

HVA, homovanillic acid; 5HIAA, 5-hydroxyindolacetic acid (serotonin end-metabolite).

*Reference values and ranges see ref. [33].

**Reference values see ref. [34].

To understand the underlying inheritance, we performed whole exome sequencing of the two ASD cases with isolated low brain serotonin turnover and carrying the PMAT-A138T or D326E mutation, plus their unaffected parents and siblings. The data were filtered for non-synonymous amino acid substitutions within published serotonin-related and/or autism-associated candidate genes (Additional file 7: Tables S4 and S5). Furthermore, using NCBI population diversity, non-synonymous mutations with an allele frequency <1 were excluded. We used a hypothetical “genetic-accumulation-model” with all the non-synonymous findings from the serotonin-related and/or autism-associated candidate gene lists, according to Additional file 7: Tables S4 and S5. Alterations with an allele frequency <1 were amalgamated (“total gene hits”) in the two affected patients plus their unaffected family members (parents and sisters) (Additional file 3: Figure S4). Using such limited analyses, the cumulative genetic burden of the two ASD affected patients showed a tendency towards more “serotonin gene hits”, indicating alterations in mainly serotonin homeostasis compared to the other family members (Additional file 3: Figure S4).

## Discussion

In contrast to an equal distribution of the previously described *SLC6A4* p.G56A mutation among the ASD and control population, in this study we identified and linked three novel heterozygous PMAT mutations to ASD and demonstrate *in vitro* functional loss of serotonin and dopamine re-uptake in two mutations. The most frequently encountered mutation p.A138T in *SLC29A4*/PMAT could be identified among four unrelated families although we did not investigate the possibility of a common founder mutation. Our previous study showed that treatment with 5-OH-tryptophan and carbidopa in one PDD-NOS-affected patient with a serotonin re-uptake transporter mutation (SERT-G56A) and isolated low 5HIAA in the CSF resulted in clinical improvement and normalization of the brain serotonin turnover [21]. In this perspective, it would be interesting to investigate whether all ASD-affected patients with serotonin re-uptake transporter mutations, such as SERT and PMAT, in combination with low brain serotonin turnover would benefit from 5-hydroxytryptophan and carbidopa treatment. If serotonin abnormalities due to hyperserotonemia, isolated low 5HIAA in the CSF, and functional serotonin re-uptake transporter mutations are essential for the etiology of ASD, the possibility for new and improved therapies may be easier to identify. The impact of the newly identified PMAT mutations leading to loss of function of the PMAT protein *in vitro* remains to be discussed with respect to changes *in vivo* regarding brain and peripheral serotonin as well as the impact on dopamine metabolism. After synaptic release of serotonin, clearance of serotonin from the synaptic cleft is mediated by the combined action of membrane transporter proteins such as SERT, PMAT, and the organic cation transporter 3 (OCT3) [35]. As described in several publications over the years, SERT is a high-affinity low-capacity transporter responsible for serotonin re-uptake back into the pre-synaptic serotoninergic neuron, where it can be recycled again into pre-synaptic vesicles [35]. In contrast to SERT, PMAT has been found to be located in the non-serotoninergic neurons surrounding the serotoninergic synaps and represents a low-affinity high-capacity transporter [35,36]. Rat brain studies showed that *SLC29A4*/PMAT-mRNA was found in various neuron subtypes, including glutamatergic, GABAergic, and cholinergic neurons throughout the brain and almost not in monoaminergic nuclei. These neurons participate in neuronal circuitries implicated in locomotion, associative and spatial memory, and reward-related learning [37].

After uptake of serotonin from the synaptic space by PMAT-expressing neurons, serotonin will probably be catabolized to 5HIAA within these neurons. In the adult human and mouse brain, *SLC29A4*/PMAT-mRNA and protein have been demonstrated to be broadly expressed and abundant in forebrain cortex, olfactory tubercle, hippocampus, cerebellum, and epithelial cells of the choroid plexus [25,36]. The study by Dahlin and coworkers has shown that PMAT is co-expressed throughout most brain regions together with the high affinity serotonin transporter (SERT) and the dopamine transporter (DAT), but is also found in certain sites that receive monoamine innervations but lack significant expression of SERT or DAT [36]. In the event of PMAT mutations with loss of function, such as PMAT-A138T and PMAT-D326E (Figure 2a–c), we suspect that serotonin and dopamine clearance become particularly compromised within those synaptic sites lacking SERT and DAT expression. The reduced monoamine clearance from these sites explains the down-regulated catabolism of monoamines, while the presence of higher monoamine concentrations within the synaptic space may exert a negative feedback inhibition through auto-receptors of local serotonin synthesis within pre-synaptic nerve terminals. However, more studies on the human brain are required to provide further insight into basic pathways of monoamine homeostasis in several brain regions among healthy controls and the alterations among patients with ASD suffering from SERT and/or PMAT mutations.

In human post-mortem brain tissue from children with ASD, consistent findings were focal patches of abnormal laminar cyto-architecture and cortical disorganization of neurons in layers 4 and 5 of the prefrontal and temporal cortex, whereas variable findings were heterotopias, loss of cerebellar Purkinje cells, and a reduced number of synapses [38,39]. In addition to its known neuro-modulatory role as a neurotransmitter, serotonin acts as an essential signaling molecule during brain development enabling normal migration, cerebral cortex differentiation, and formation of neuronal networks [40,41].

Whole blood serotonin and platelet serotonin content are increased in about 25 to 30% of the ASD population and their first-degree relatives. Because the fetal blood–brain barrier during pregnancy is not yet fully formed, the fetal brain will be exposed to high serotonin levels, leading through a negative-feedback mechanism to a loss of serotonin neurons and a limited outgrowth of their terminals. This hypothesis has been confirmed by rat studies using the serotonin agonist 5-methoxytryptamine between gestational days 12 until postnatal day 20 [42].

Incomplete ASD-behavior has also been observed in more specific genetic rodent models disrupting early serotonin neuron development, driven by the *pet-1 ETS* gene or *Celf6*-gene, which resulted in reduced serotonin levels [43,44]. Likewise, ASD-like behavior has been observed in both *SLC6A4* gene (SERT) knockout mice and the *SLC6A4* Ala56 high activity SERT variant; both latter models finally result in decreased serotoninergic signal transmission. A more complete phenotype of ASD could be observed in *Tph2*^-/-^ mice via a null mutation of the gene for TPH2, the rate-limiting enzyme for serotonin production. In addition to these genetic models, injection of the serotoninergic neurotoxin, 5,7-dihydroxytryptamine into the bilateral medial forebrain bundle of mice at birth also reproduced similar results [41].

These rodent studies underline that during the pre- and post-natal period a tight regulation of serotonin signaling between strict boundaries is required to prevent ASD, because either prenatal exposure to high serotonin through the placenta or hypo-serotonin conditions perturb early development of brainstem serotonin neurons and outgrowth of their terminals, where serotonin fails to act as a signaling molecule for normal brain development. PET-scan studies in young children with ASD have confirmed loss of serotonin production in cerebral cortex and subcortical structures [14,18,19].

PMAT is widely expressed in all brain areas and skeletal muscles. In contrast to the high-affinity low-capacity serotonin membrane transporter SERT, PMAT is a low-affinity high-capacity transporter for biogenic monoamines and organic cations. Both SERT and PMAT transporters cooperate in recycling and degradation of serotonin from synapses to terminate serotonin signaling. In addition to its wide expression in the brain, PMAT is also expressed at the apical side of choroid plexus epithelial cells (CSF-face), where it functions as a major transporter of biogenic monoamines and xenobiotic cations from cerebrospinal fluid into the choroid epithelial cells. In *Slc29a4*^*-/-*^ mice, loss of PMAT expression at the apical surface of choroid plexus was found to impair monoamine and organic cation uptake from the CSF compartment [45]. Like in *Slc6a4*^-/-^ knockout mice, we speculate that loss of PMAT function due to mutations or deletions of the encoding gene will diminish serotonin reuptake from synapses and choroid plexus-mediated clearance of serotonin from the CSF. During the prenatal period, this may result in a hyper-serotonin condition with negative feedback inhibition on development of brainstem serotonin neurons and outgrowth of their axonal branches. At later stages, the loss of PMAT function further exerts a negative feedback inhibition through pre-synaptic auto-receptors on local TPH2-mediated serotonin synthesis, which ultimately leads to long-term serotonin depletion in subcortical and cortical areas, as outlined above.

However, the real situation appears more complex for the following reasons: i) although PMAT mutations are linked to the ASD population, these mutations in ASD subjects were inherited from unaffected parents and ii) despite demonstration of reduced serotonin and dopamine re-uptake by cell cultures expressing two PMAT mutants *in vitro*, CSF analysis did show low serotonin metabolites in some of these patients but not in all cases. The genome sequencing data indicated that two autistic subjects with PMAT mutations and functional loss *in vitro* associated with low 5HIAA in CSF, had the highest burden of abnormal serotonin homeostasis genes, as compared to their siblings and parents, whereas the other autism-associated candidate genes did not show a higher accumulation for the ASD patients in comparison to their siblings and parents. In the identical twins with the PMAT mutation whose CSF 5HIAA levels were normal, genome sequencing of serotonin homeostasis genes has not been performed but might improve our understanding for the normal CSF levels. The fact that PMAT mutations with functional loss of monoamine re-uptake *in vitro* is not invariably associated with reduced serotonin metabolite levels in spinal fluid would suggest that these PMAT mutations exert a more important pathophysiological role in the prenatal period of brain development, as outlined above.

## Conclusions

In summary, with our findings we were able to link novel mutations in *SLC29A4* to ASD. In addition to analysis of serotonin re-uptake transporter genes, exome sequencing data in ASD and their first-degree relatives should focus on serotonin homeostasis genes to provide a better understanding of the mechanisms of aberrant serotonin signaling in ASD. This strategy might offer the development of new tools to provide a better pharmacological approach and new therapeutic targets.

## Abbreviations

AADC: Aromatic amino acid decarboxylase; ASD: Autism spectrum disorder; CSF: Cerebrospinal fluid; DAT: Dopamine transporter; GFRP (gene *GCHFR*): GTP cyclohydrolase I feedback regulatory protein; 5HIAA: 5-hydroxyindolacetic acid; PDD-NOS: Pervasive developmental disorder – not otherwise specified; PMAT: Plasma membrane monoamine transporter; SERT: Serotonin re-uptake transporter; TPH2 (gene *Tph2*): Tryptophan hydroxylase 2; VNTR: Variable number tandem repeat; YFP: Yellow fluorescence protein.

## Competing interests

The authors declare no conflict of interest.

## Authors’ contributions

Study design, data interpretation and writing of the manuscript were carried out by DA, VR, EG, and BT, while DA performed the majority of the experiments including DNA sequencing (*SLC6A4*, *SLC29A4* and *GCHFR)*, genotyping analysis (5-HTTLPR and STin2 VNTR) and site-directed mutagenesis. VR, PP, CN, and LVM collected ASD-affected patients (Liège and Paris cohort), and VR wrote case reports for the two ASD subjects MT and PL. CB, VR, and DM assisted with sequence analyses and site-directed mutagenesis, RB performed whole exome sequencing. HTBH and JW carried out the functional analyses of PMAT mutants, and EB, SW and EG collected ASD-affected patients (Zürich cohort). All co-authors commented on the manuscript. All authors read and approved the final manuscript.

## Supplementary Material

Additional file 1 **Supplementary Patient Information, Materials and Methods.** Contains supplementary information on patients (case reports), methods, and the text for Figures S1 to S4. Click here for file

Additional file 2: Tables S1, S2, and S3 Contains supplementary information on Tables S1, S2, and S3. Click here for file

Additional file 3: Figure S4 Adamsen et al. contains supplementary Figure S4. Click here for file

Additional file 4: Figure S1 Adamsen et al. contains supplementary Figure S1. Click here for file

Additional file 5: Figure S2 Adamsen et al. contains supplementary Figure S2. Click here for file

Additional file 6: Figure S3 Adamsen et al. contains supplementary Figure S3. Click here for file

Additional file 7: Tables S4 and S5 Contains supplementary information on Tables S4 and S5. Click here for file
