# Supplementary material for: Autism spectrum disorder associated with low serotonin in CSF and mutations in the SLC29A4 plasma membrane monoamine transporter (PMAT) gene
Source: Mol Autism. 2014 Aug 13;5:43. doi: 10.1186/2040-2392-5-43 (PMC4370364; doi:10.1186/2040-2392-5-43)
Supplement: Additional file 1 — Supplementary Patient Information, Materials and Methods. Contains supplementary information on patients (case reports), methods, and the text for Figures S1 to S4. [file 2040-2392-5-43-S1.doc]

## Supplementary Patient Information, Materials and Methods, AND FIGURE TEXT

***Subjects***

A total of 248 ASD-affected patients collected from three cohorts (97 from Liège, 71 from Paris and 80 from Zürich) have taken part in this study plus additional 394 control subjects from an *in house* collection representing all ages, both sex and with a Caucasian background. The affected patients were diagnosed with ASD, by their local clinicians in Liège, Paris and Zürich, according to the Diagnostic and Statistical Manual of Mental Disorders, Fourth Edition (DSM-IV), the ICD-10 Classification of Mental and Behavioral Disorders (<http://www.who.int/classifications/icd/en/>), as well as ADI-R and ADOS. CSF analyses were performed in 97 subjects (Liège-cohort) according to approval by the ethics committee at Liege University in a study protocol for ASD patients to detect cerebral folate deficiency and disturbed neurotransmitter metabolism (study number B70720083916 approved on July 24^th^ 2008 by the Comité d´Ethique Hospitalo-Facultaire Universitaire de Liège with the use of full oral information and written informed consent forms, signed by the patients and/or their parents and filed for future documentation). Genomic DNA for genetic analysis (SLC29A4, SLC6A4, 5-HTTLPR, Stin2 VNTR and GCHFR) of all involved subjects was isolated from blood samples or saliva after obtaining informed consent from them or their parents. For methods of 5HIAA-quantification in CSF see reference given in **Table 1**.

**Case report (MT)**

A now 11 year old boy was born after an uncomplicated pregnancy, delivery and neonatal period. Both parents are known to suffer from anxiety disorder since childhood but do not have further health problems. His father works as a bank director and his mother used to work as a nurse in an infirmary. For his anxiety disorder paroxetine, a SSRI drug was prescribed to the father. The mother takes sertraline (an [antidepressant](http://en.wikipedia.org/wiki/Antidepressant) of the [selective serotonin re-uptake inhibitor](http://en.wikipedia.org/wiki/Selective_serotonin_reuptake_inhibitor) (SSRI) class). A 5 year old younger sister is healthy. The psychomotor and speech development were normal in the patient. However, the parents noticed that he did not show a normal imitative play and from early infancy, he suffered from sleeping problems due to anxiety. When he started kindergarten at the age of 2 ½ years he was described as a nervous child who did not seek contact with other children, was found to be “egocentric”, did not play with fantasy, did not like changes and responded with anxiety, but showed restrictive interests in busses and trains. He had few stereotypic movements like putting his hands in front of his mouth with an anxious expression. He had an extreme phobia for insects. From the age of 5 years he was seen by a child psychiatrist who suspected Asperger’s syndrome with attention deficit disorder and prescribed methylphenidate in combination with risperidone at night. Formal testing and observation at home and in school at the age of nearly 9 years was performed by the centre for autism, using DSM-IV criteria, neuropsychological tests, and evaluation of socio-emotional comprehension, Childhood Autism Rating Scale (CARS), ADI-R and ADOS, and the Childhood Apperception Test (CAP). His physical and neurologic examinations were completely normal. In conclusion, he was found to fulfill all criteria for Asperger’s syndrome with a CARS score of 39.5, indicating moderate to severe autism. After informed consent was given, laboratory investigations at the age of 9½ years revealed normal routine tests including amino-acid levels. The only abnormality observed was a moderately diminished serum gamma-tocopherol. In addition, spinal fluid analysis showed a low serotonin metabolite 5HIAA at 65 nmol/L (normal range: 88-178 ^1^), a normal HVA concentration at 389 nmol/L (reference: 144-801) with an elevated HVA/5HIAA ratio at 6 (normal value 1.5-3.5). Spinal fluid concentrations for L-dopa, 5-hydroxytryptophane, pterins and L-N^5^-methyltetrahydrofolate were normal. The whole blood serotonin level was slightly elevated at 287 ng/ml (normal range 50-200 ^2^) and platelet serotonin content was elevated at 1041 ng/10^9^ platelets ( normal range: 125-500 ^2^).

**Case report (PL)**

A now 8 year old boy was born by caesarean delivery after a pregnancy of 37 weeks, complicated by maternal hypertension and gestational diabetes. His birth weight was 3.7 kg. His parents were healthy and non-consanguineous. He had an 11 month older sister in good health. After a normal neonatal period he was described as an inactive baby, never crying and could be put anywhere. Developmental motor milestones were attained normally but as he started toe-walking from the age of 2 years, he would frequently fall. From the age of 2 years, he started to have severe eating problems and would only eat biscuits, chicken and French fries. During his first two years, he did not respond to his name, never pointed or attracted joint attention; he never waved good-bye and showed restrictive interests with absent imitative play. From the age of 3 years, he developed stereotypic patterns like running in circles, turning light switches on and off, balancing movements and head banging against the floor. Because of serous middle ear effusions and suspected hearing loss at the age of two years, bilateral tympanic drains were placed but postoperatively there was no improvement of his response to external stimuli and speech did not develop. He could utter single word fragments using syllables from the age of 3 years and developmental dysphasia was suspected. From the age of 4½ years he was transferred from kindergarten to a special school for mentally handicapped children. The Autism Diagnostic Interview for parents (ADI-R) revealed that he fulfilled all criteria of infantile autism between the age of 4 and 5 years. Physical examination did not show any dysmorphic features and neurological status was completely normal. Twenty-four EEG monitoring and brain MRI were found normal. Laboratory investigations at the age of 6 years revealed normal CSF cell counts, protein and glucose levels. Spinal fluid analysis showed diminished concentrations for the serotonin end-metabolite 5HIAA but normal levels for the dopamine end-metabolite HVA, intermediate metabolites L-dopa, 5-hydroxytryptophane, pterines and L-N^5^-methyl- tetrahydrofolate. Serum amino-acids were normal including L-tryptophane. The whole blood serotonin level was slightly elevated at 221 ng/ml (normal range 50-200 ^2^) and platelet serotonin content was elevated at 675 ng/10^9^ platelets (normal range: 125-500 ^2^). Biochemical blood examination revealed slight deficiencies for serum and red blood cell folate content, as well as vitamin C, D, gamma-tocopherol, beta-carotene, zinc and selenium. He received vitamin and trace element supplements to correct these alimentary deficiencies.

From the age of 6 years he visited a special institute for autistic children and received re-education by the **TEACCH** method (**T**reatment and **E**ducation of **A**utistic and **C**ommunication related handicapped **CH**ildren) and using pictogrammes. Follow-up assessment at the age of 7 4/12 years using the **ADI-R**, **A**utism **D**iagnostic **O**bservation **S**cale (**ADOS**) as well as observation at home and school, revealed moderate improvement with respect to non-verbal communication and behavior, so that he now fulfilled the criteria for atypical autism (PDD-NOS).

Screening of mutations in human GFRP

The following intronic primers were used to screen the human GFRP/*GCHFR* gene (OMIM: 602437; [ENST00000260447](http://www.ensembl.org/Homo_sapiens/Transcript/Summary?db=core;g=ENSG00000137880;r=15:41056218-41060084;t=ENST00000260447)) (Exon 1: GFRP5: 5’-TCCTCCCCGCACTCCACCTT-3’ (forward) and GFRP6: 5’-CTCCTGTCCTACCTGTCGGC-3’ (reverse), Exon 2: GFRP7, 5’-TCAAACTC CTCCCACCTCAA-3 (forward) and GFRP8: 5’-TAGAAGCAGGGAGCAGGGAG-3’ (reverse), Exon 3: GFRP9, 5’-CTGGGACGCTGGGGAACCTG-3 (forward) and GFRP10: 5’-GTCAAGC AGTGGGAGAGGGC-3’ (reverse)). PCR reactions were performed in a final volume of 25μl containing 1μl of genomic DNA template (100 ng), 1μl of each primer (5 μM), 0.5μl 10 mM deoxyribonucleoside triphosphates (dNTPs), 0.5μl Hot FirePol Hotstart DNA polymerase (Solis Biodyne), 1.5μl 25 mM MgCl_2_, 2.5μl 10x reaction buffer (Mg^2+^ free from Solis Biodyne) and 5μl 5 x Q-solution (Qiagen). The PCR reactions were performed in a GeneAmp PCR System 9700 from Applied Biosystems using standard thermal cycling: hot start activation at 95^o^C for 15 minutes, followed by 35 cycles of denaturation 95°C for 60 seconds, annealing 54°C for 45 seconds and extension at 72°C for 30 seconds, a final extension was performed at 72°C for 10 minutes and termination at 4^o^C. The same forward and reverse primers as mentioned above were used to sequence the amplified PCR products with BigDye Terminator v1.1 Cycle Sequencing Kit (Applied Biosystems). Sequencing PCR was performed in a 10 μl reaction mixture consisting of 0.5 μl PCR product, 0.8 μl primer (5 μM), 1.5 μl BigDye and 1.25 μl 5 x sequencing buffer (Both BigDye and 5 x sequencing buffer were from the BigDye Terminator v1.1 Cycle Sequencing Kit) using standard thermal cycling: (activation at 96^o^C for 1 min, followed by 25 cycles of 96^o^C for 10 seconds, 54^o^C for 5 seconds, 60^o^C for 1.5 minutes and termination at 4^o^C). After end run 15 μl H_2_O was added to the sequencing reactions before purified by gel filtration (using MultiScreen HV 96-well filter plates (Millipore) with Sephadex G-50 (GE Healthcare)) and analyzed on a 3130*xl* Genetic Analyzer (Applied Biosystems). Sequencing results were compared with the reference sequence (Transcript ID: [ENST00000260447](http://www.ensembl.org/Homo_sapiens/Transcript/Summary?db=core;g=ENSG00000137880;r=15:41056218-41060084;t=ENST00000260447) or accession number [NM_005258.2](http://www.ncbi.nlm.nih.gov/nuccore/NM_005258.2)) of the *GCHFR* gene.

***Search strategy and selection criteria for whole exome sequencing analysis***

To analyze the whole exome sequencing data, we focused on candidate genes published to be involved in serotonin metabolism/signaling as well as candidate genes involved in ASD. In order to find these candidate genes we first made a literature searched in Pubmed with the term: “serotonin” in combination with “synthesis”, “metabolism”, “transporter”, “receptor”, “transcription factor”, “regulation”. This was followed by a literature searched with the term: “autism” in combination with “candidate genes”, “genes” and “genetics”. These Pubmed search strategies led to the generation of two lists: one containing the candidate genes of interest published to be involved in serotonin metabolism/signaling (see **Supplementary Table S4**) and one with the candidate genes of interest involved in autism, ASDs and ASD-related syndromes (see **Supplementary Table S5**).

**Supplementary Figure Text**

**Supplementary Figure S1: Sanger DNA-sequencing and mutation identification.** Heterozygous non-synonymous alterations were identified in the *SLC29A4* gene encoding PMAT in various ASD patients, shown here for AH, MT, and PL, carrying mutations c.86A>G/p.D29G, c.412G>A/p.A138T, and c.978T>G/p.D326E, respectively (see also text and supplementary information on patients).

**Supplementary Figure S2: Position of newly identified alternations in the secondary structure of human PMAT**

In humans, the *SLC29A4* gene encodes a 530 amino acid membrane protein with 11 predicted membrane-spanning domains, an intracellular N-terminus and an extracellular C-terminus. The three identified mutations PMAT-D29G, PMAT-A138T and PMAT-D326E are marked by filled red circles.

**Supplementary Figure S3: Multiple sequence alignment of PMAT from various species**

D29, A138 and D326 in hPMAT and their corresponding residues in PMAT orthologs are highlighted in red boxes (z: zebrafish; r: rat; m: mouse; c: cattle; ma: marmoset; rh: rhesus monkey; h: human).

**Supplementary Figure S4: Cumulative genetic burden using a “genetic-accumulation-model”**

When all the non-synonymous findings from the serotonin-related (in red) and autism-associated (in gray) candidate gene lists with an allele frequency <1 are weighed, the cumulative genetic burden shown more “serotonin gene hits” in the two index patients compared to their healthy family members. The two VNTR polymorphisms (5-HTTLPR and STin2VNTR) in the *SLC6A4* gene are not included in the cumulative genetic burden as their functions have been found to be complex and controversial. In bold are the genes which are mutated in patients MT and PL. Further information or pathogenicity on all these mutations (except for PMAT) can found in the references given in the Supplementary Tables S4 and S5. Full-triangle, homozygous; half-triangle, heterozygous; empty-triangle, wild-type; n.d., not determined. L, S: the [promoter](http://en.wikipedia.org/wiki/Promotor_(biology)) region of the *SLC6A4* gene contains a [polymorphism](http://en.wikipedia.org/wiki/Polymorphism_(biology)) with a "short" (S) variation with 14 repeats and a "long" (L) variation with 16 repeats in the 5-HTT-linked polymorphic region.

**References**

1. Blau N, Bonafe L, Blaskovics ME. Disorders of phenylalanine and tetrahydrobiopterin metabolism. *Physician's Guide to the Laboratory Diagnosis of Metabolic Diseases* 2003; **Springer 2nd Edition, Blau, N., Duran, M., Blaskovics, M.E., Gibson, K.M. (eds):** 89-106.

2. Burtis CA, Ashwood ER, Tietz A. Fundamentals of Clinical Chemistry. *WB Saunders, Philadelphia* 2001; **5th Edition**.

3. Abecasis GR, Altshuler D, Auton A, Brooks LD, Durbin RM, Gibbs RA *et al.* A map of human genome variation from population-scale sequencing. *Nature* 2010; **467**(7319)**:** 1061-1073.

4. Adamsen D, Meili D, Blau N, Thony B, Ramaekers V. Autism associated with low 5-hydroxyindolacetic acid in CSF and the heterozygous SLC6A4 gene Gly56Ala plus 5-HTTLPR L/L promoter variants. *Mol Genet Metab* 2011; **102**(3)**:** 368-373.

5. Zhou M, Xia L, Engel K, Wang J. Molecular determinants of substrate selectivity of a novel organic cation transporter (PMAT) in the SLC29 family. *J Biol Chem* 2007; **282**(5)**:** 3188-3195.

6. Engel K, Zhou M, Wang J. Identification and characterization of a novel monoamine transporter in the human brain. *J Biol Chem* 2004; **279**(48)**:** 50042-50049.
