# Supplementary material for: Autism spectrum disorder associated with low serotonin in CSF and mutations in the SLC29A4 plasma membrane monoamine transporter (PMAT) gene
Source: Mol Autism. 2014 Aug 13;5:43. doi: 10.1186/2040-2392-5-43 (PMC4370364; doi:10.1186/2040-2392-5-43)
Supplement: Additional file 2: Tables S1, S2, and S3 — Contains supplementary information on Tables S1, S2, and S3. [file 2040-2392-5-43-S2.doc]

**Supplementary Table S1:**

**Primers used for PCR-amplification and DNA sequencing of the human *SLC29A4* gene**

**_________________________________________________________________________**

**­­­­­­­­­­Region Primer Sequence (forward and reverse) Product size (bp)**

Exon 2 5’-ATGGATCCCTCAGCCTTCT-3’ 285

5’-ACAGCCCAGCCTGCTTT-3’

Exon 3 5’-GGTGACTGTAGCCATGCGT-3’ 242 5’-CAGGCCATGATGGAGGAT-3’

Exon 4 5’-CAGGAGTCAGTGCAGGGG-3’ 253 5’-GGAAACTGGGTGAGTGGGT-3’

Exon 5 5’-GACTCTGCAGGAGGGGC-3’ 263

5’-GATGGGGTCGGAAGTGG-3’

Exon 6 5’-TCTCCCAAGTTGACAGGTG-3’ 198

5’-AAGAAGCTTCCAGAGCCCA-3’

Exon 7 5’-GTCTGTGTGTGGACGGGG-3’ 403

5’-GGGGACAGAGAAGGGGAC-3’

Exon 8-9 5’-CCCGTGTCTCCTGTCCTC-3’ 563

5’-CAGGGCCTCCCTTGTCAC-3’

Exon 10 5’-GGATGTGGCTAGAGGCTGT-3’ 403

5’-GTCTAGCCTGGGTTTCCTC-3’

Exon 11 5’-GTCACCGCACCTCACACC-3’ 312

5’-CACTGAGGTGGGGACAGG-3’

**Supplementary Table S2:**

**Serotonin re-uptake transporter (SERT/*SLC6A4*) and plasma membrane monoamine transporter (PMAT/*SLC29A4*) mutations in 248 ASD patients diagnosed with normal, unknown or isolated low 5HIAA in the CSF**

**___________________________________________________________________________**

**Gene Protein Heterozygous mutation Patient / Cohort ^a^ (ASD) 5HIAA in CSF (nmol/L)**

__________________________________________________________________________________________

*SLC6A4* SERT p.G56A CM / L (PDD-NOS) Low

*SLC6A4* SERT p.G56A PL / L (PDD-NOS) ^b^ Low (Table 1)

*SLC6A4* SERT p.G56A VE / L (Asperger’s) Normal but in low range

*SLC6A4* SERT p.G56A ARN / P (PDD-NOS) Unknown

*SLC6A4* SERT p.G56A CAT / P (Kanner) Unknown

*SLC29A4* PMAT p.D29G AH / L (PDD-NOS) Normal

*SLC29A4* PMAT p.D29G ACI / P (Kanner) Unknown

*SLC29A4* PMAT p.A138T MT / L (Asperger’s) Low (Table 1)

*SLC29A4* PMAT p.A138T EGN / L (Kanner) ^c^ Normal

*SLC29A4* PMAT p.A138T EGA / L (Kanner) ^c^ Normal

*SLC29A4* PMAT p.A138T A33 / Z (atypical ASD) Unknown

*SLC29A4* PMAT p.A138T A95 / Z (Asperger’s) Unknown

*SLC29A4* PMAT p.D326E PL / L (PDD-NOS) ^b^ Low (Table 1)

__________________________________________________________________________

^a^ Cohort: Liège (L), Paris (P), Zürich (Z).

^b^ Patient PL was found both to carry a heterozygous mutation within the SERT/*SLC6A4* gene (p.G56A) and the PMAT/*SLC29A4* gene (p.D326E).

^c^ Patient EGN and EGA are identical twin brothers from first-grade cousins of Tunisian descent.

**Supplementary Table S3:**

**MutationTaster and PolyPhen2 prediction of possible impact of amino acid substitution on PMAT protein function**

| **Mutation** | **MutationTaster Prediction ^a^** | **MutationTaster Prediction ^b^** |
| --- | --- | --- |
| **p.D29G** | protein features (might be) affected | possibly damaging (score 0.780) |
| **p.A138T** | protein features might be affected | benign (score 0.003) |
| **p.D326E** | protein features (might be) affected | probably damaging (score 0.960) |

^a^ MutationTaster (http://www.mutationtaster.org) is an application for rapid evaluation of the disease-causing potential of DNA sequence alterations, and analyses comprise, among other features, evolutionary conservation (Nat Methods 2010;7:575-576).

^b^ PolyPhen2 (Polymorphism Phenotyping v2; http://genetics.bwh.harvard.edu/pph2/) is a tool which predicts possible impact of an amino acid substitution on the structure and function of a human protein and utilizes multiple alignments of 45 vertebrate genomes (Nat Methods 2010;7:248-249).
