# Supplementary material for: Autism spectrum disorder associated with low serotonin in CSF and mutations in the SLC29A4 plasma membrane monoamine transporter (PMAT) gene
Source: Mol Autism. 2014 Aug 13;5:43. doi: 10.1186/2040-2392-5-43 (PMC4370364; doi:10.1186/2040-2392-5-43)
Supplement: Additional file 3: Figure S4 — Adamsen et al. contains supplementary Figure S4. [file 2040-2392-5-43-S3.ppt]

## Slide 1
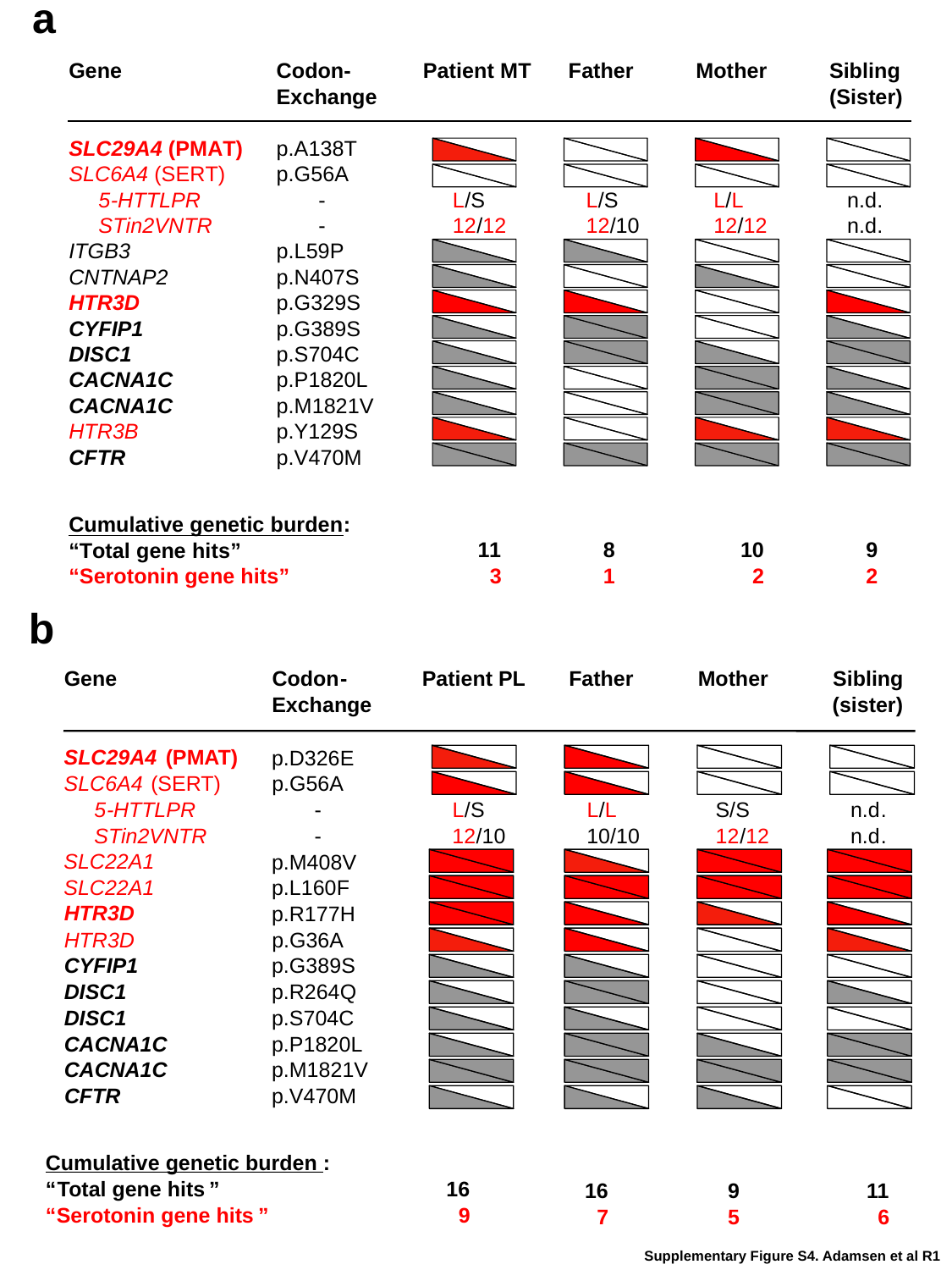

a
b
Gene
Codon
-
Patient PL
Father
Mother
Sibling
Exchange
(sister)
(PMAT)
SLC29A4
p.D326E
SLC6A4
(SERT)
p.G56A
5
-
HTTLPR
-
L
/S
L
/
L
S/S
n.d
.
STin2VNTR
-
12
/10
10/10
12
/
12
n.d
.
SLC22A1
p.M408V
SLC22A1
p.L160F
HTR3D
p.R177H
p.G36A
HTR3D
CYFIP1
p.G389S
DISC1
p.R264Q
DISC1
p.S704C
CACNA1C
p.P1820L
CACNA1C
p.M1821V
CFTR
p.V470M
Cumulative genetic burden
:
“
Total gene hits
”
16
16
9
11
“
Serotonin gene hits
”
9
7
5
6
Supplementary Figure S4. Adamsen et al R1
