# Supplementary figures and images for: Autism spectrum disorder associated with low serotonin in CSF and mutations in the SLC29A4 plasma membrane monoamine transporter (PMAT) gene
Source: Mol Autism. 2014 Aug 13;5:43. doi: 10.1186/2040-2392-5-43 (PMC4370364; doi:10.1186/2040-2392-5-43)

## Slide 1
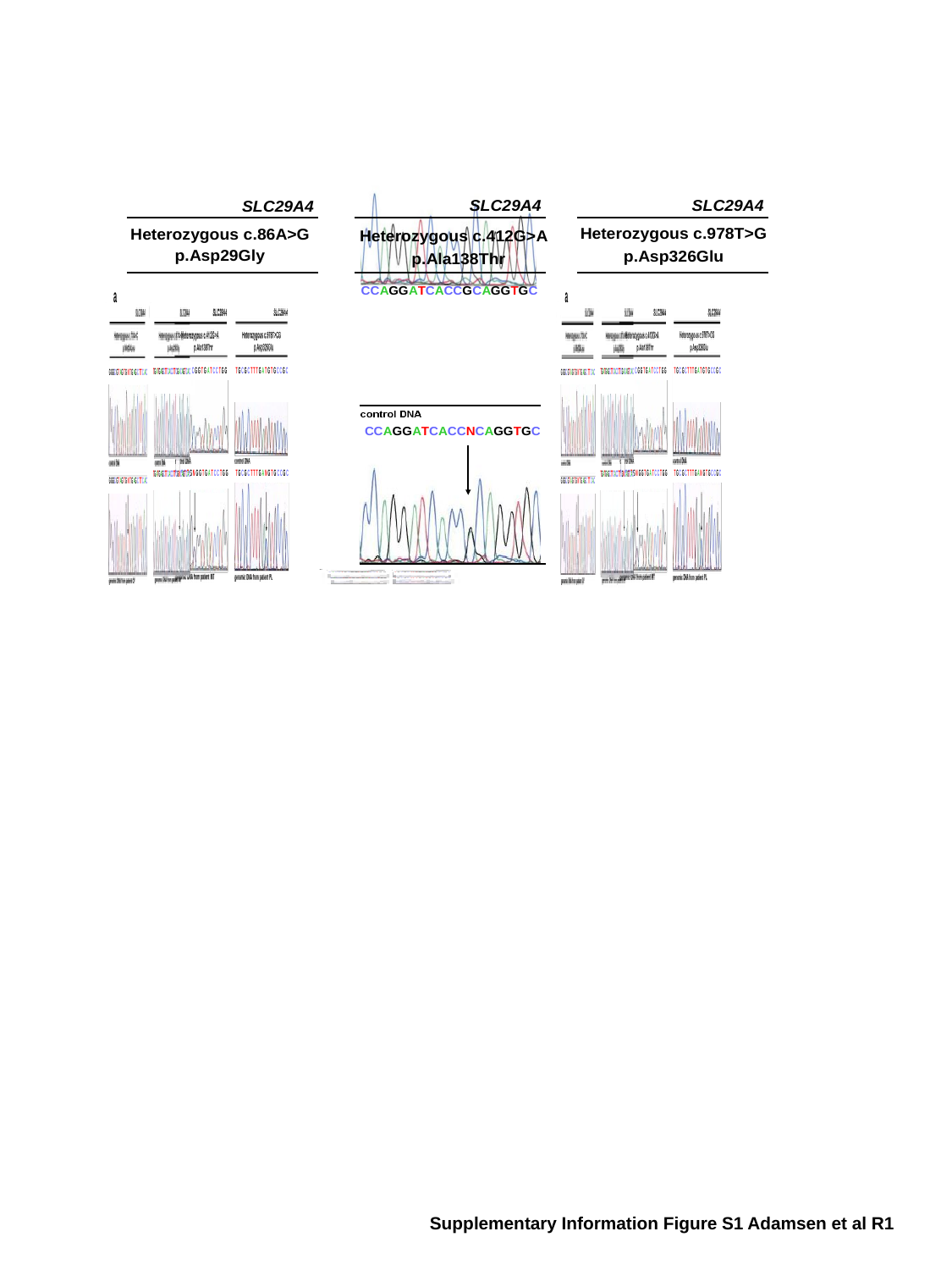

Supplementary Information Figure S1 Adamsen et al R1

Supplement: Additional file 4: Figure S1 — Adamsen et al. contains supplementary Figure S1. [file 2040-2392-5-43-S4.ppt]

## Slide 1
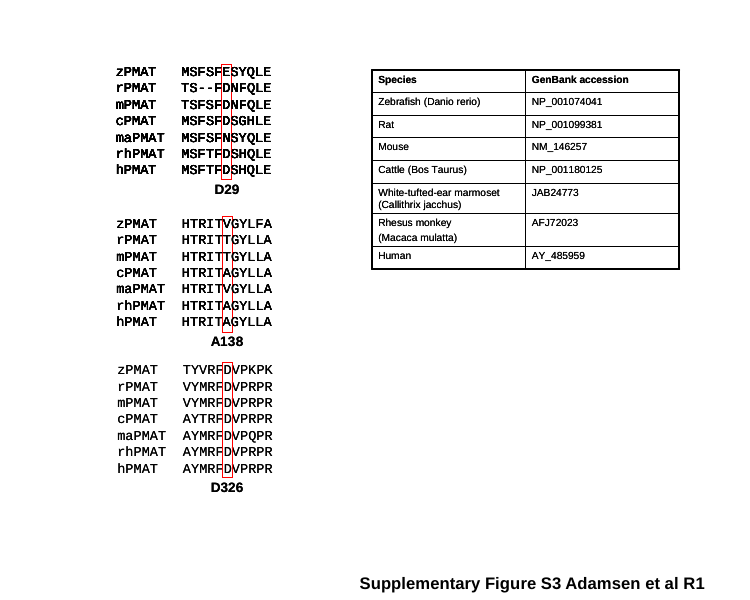

Supplementary Figure S3 Adamsen et al R1

Supplement: Additional file 6: Figure S3 — Adamsen et al. contains supplementary Figure S3. [file 2040-2392-5-43-S6.ppt]
