# Supplementary material for: Autism spectrum disorder associated with low serotonin in CSF and mutations in the SLC29A4 plasma membrane monoamine transporter (PMAT) gene
Source: Mol Autism. 2014 Aug 13;5:43. doi: 10.1186/2040-2392-5-43 (PMC4370364; doi:10.1186/2040-2392-5-43)
Supplement: Additional file 5: Figure S2 — Adamsen et al. contains supplementary Figure S2. [file 2040-2392-5-43-S5.ppt]

## Slide 1
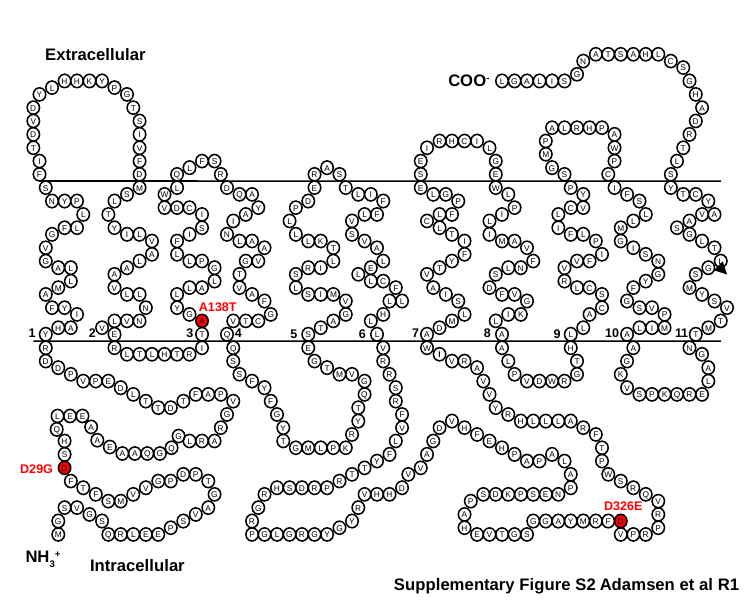

Extracellular
A
T
S
A
H
L
N
C
S
COO-
G
H
H
K
Y
L
G
A
L
I
S
G
L
P
Y
G
H
D
T
A
V
S
D
A
L
R
H
P
D
I
A
R
R
H
C
I
P
T
V
I
L
W
T
M
I
F
F
S
E
G
P
L
L
A
G
F
D
Q
R
R
S
S
E
S
C
S
S
M
L
D
E
T
E
W
P
I
Y
S
W
Q
A
L
I
L
G
L
Y
F
T
C
N
Y
P
L
D
F
P
S
Y
V
D
C
Y
P
P
C
V
L
T
I
A
L
F
L
F
I
L
L
V
A
I
L
V
C
L
L
A
F
L
Y
S
L
I
M
S
G
I
L
I
N
L
S
T
I
F
L
G
V
F
L
A
L
K
V
I
M
A
P
G
L
V
A
T
A
V
I
T
A
L
F
I
S
G
L
L
P
G
V
L
L
Y
F
V
F
N
L
A
L
A
G
R
I
E
T
L
N
V
G
A
T
S
L
V
S
G
S
L
L
L
C
R
Y
M
V
L
A
V
L
F
A
D
L
C
F
M
A
L
L
L
A
S
I
M
I
F
V
S
Y
F
V
L
L
S
G
G
S
A138T
F
Y
N
Y
C
S
V
V
I
G
G
G
H
L
I
K
A
P
L
V
N
A
V
T
C
A
L
M
L
T
1
H
A
V
T
7
D
8
L
10
L
I
M
11
M
2
3
4
5
6
9
Y
E
T
Q
S
L
A
A
L
A
T
R
R
I
Q
E
V
W
A
H
A
N
L
T
L
H
T
R
I
G
D
S
G
R
V
R
L
T
G
D
T
A
A
P
S
M
V
R
P
G
K
V
P
E
F
G
V
V
D
W
R
L
D
Y
S
V
L
F
A
P
Q
V
S
P
K
Q
R
E
T
T
V
F
R
T
D
T
Y
G
G
F
R
L
E
E
Y
V
H
L
L
L
A
A
R
Y
V
D
H
R
Q
R
F
F
G
A
H
L
R
A
T
L
G
E
E
Q
G
M
L
P
K
H
T
A
A
Q
G
S
F
A
P
A
Y
A
P
L
P
D29G
D
T
V
D
P
T
V
A
W
F
G
P
T
R
S
T
V
H
S
D
R
P
D
P
R
F
V
G
R
V
H
H
S
D
K
P
S
E
N
Q
D326E
S
M
P
V
S
V
A
G
R
G
V
A
R
G
S
S
R
Y
G
G
A
Y
M
R
F
D
P
G
H
P
M
Q
R
L
E
E
P
G
L
G
R
G
Y
E
V
T
G
S
V
P
R
NH3+
Intracellular
Supplementary Figure S2 Adamsen et al R1
