# Supplementary material for: Autism spectrum disorder associated with low serotonin in CSF and mutations in the SLC29A4 plasma membrane monoamine transporter (PMAT) gene
Source: Mol Autism. 2014 Aug 13;5:43. doi: 10.1186/2040-2392-5-43 (PMC4370364; doi:10.1186/2040-2392-5-43)
Supplement: Additional file 7: Tables S4 and S5 — Contains supplementary information on Tables S4 and S5. [file 2040-2392-5-43-S7.doc]

**Table S4: Candidate genes involved in serotonin synthesis, metabolism, transport, recycling, receptor, transcription factor and regulation**

| **Protein name** | **Gene name** | **RefSeq IDs** | **OMIM Number** | **Function of gene** | **Reference** |
| --- | --- | --- | --- | --- | --- |
| Tryptophan hydroxylase 1  Tryptophan hydroxylase 2 | *TPH1*  *TPH2* | NM_004179  NM_173353 | 191060  607478 | Serotonin synthesis  Serotonin synthesis |  |
| Aromatic L-amino acid decarboxylase (AADC)  Aldehyde dehydrogenase 2 family (mitochondrial)  Serotonin re-uptake transporter (SERT, 5-HTT)  Plasma membrane monoamine transporter (PMAT)  Organic cation transport 1 (OCT1)  Organic cation transport 3 (OCT3)  Vesicular monoamine transporter 2 (VMAT2)  Monoamine oxidase A (MAO-A)  Monoamine oxidase B (MAO-B)  Serotonin receptor 1A  Serotonin receptor 1B  Serotonin receptor 1D  Serotonin receptor 1E  Serotonin receptor 1F  Serotonin receptor 2A  Serotonin receptor 2B  Serotonin receptor 2C  Serotonin receptor 3A  Serotonin receptor 3B  Serotonin receptor 3C  Serotonin receptor 3D  Serotonin receptor 3E  Serotonin receptor 4  Serotonin receptor 5A  Serotonin receptor 5B  Serotonin receptor 6  Serotonin receptor 7  cAMP responsive element binding protein 1  S100 calcium binding protein, beta  PET-1 (Fev-pending)  NK2 homeobox 2  [LIM](http://en.wikipedia.org/wiki/LIM_domain) [homeobox](http://en.wikipedia.org/wiki/Homeobox) [transcription factor](http://en.wikipedia.org/wiki/Transcription_factor)  GATA binding protein 2  GATA binding protein 3  Achaete-scute complex homolog 1 (Drosophila)  Paired-like homeobox 2b  Homeobox B1  Homeobox B2  NK6 homeobox 1  NK6 homeobox 2  Orthodenticle homeobox 2  GLI family zinc finger 1  GLI family zinc finger 2  Fibroblast growth factor 4  Fibroblast growth factor 8  Sonic hedgehog  Smoothened homolog (Drosophila)  Gastrulation brain homeobox 2  GTP cyclohydrolase I feedback protein (GFRP)  Organic cation transporter 3 (OCT3) | *DDC*  *ALDH2*  *SLC6A4*  *SLC29A4*  *SLC22A1*  *SLC22A3*  *SLC18A2*  *MAOA*  *MAOB*  *HTR1A*  *HTR1B*  *HTR1D*  *HTR1E*  *HTR1F*  *HTR2A*  *HTR2B*  *HTR2C*  *HTR3A*  *HTR3B*  *HTR3C*  *HTR3D*  *HTR3E*  *HTR4*  *HTR5A*  *HTR5B*  *HTR6*  *HTR7*  *CREB1*  *S100β*  *FEV*  *NKX2-2*  *LMX1B*  *GATA2*  *GATA3*  *ASCL1*  *PHOX2B*  *HOXB1*  *HOXB2*  *NKX6-1*  *NKX6-2*  *OTX2*  *Gil1*  *GIL2*  *FGF4*  *FGF8*  *SHH*  *SMO*  *GBX2*  *GCHFR*  *OCT3 (SLC22A3)* | NM_000790  NM_000690  NM_001045  NM_153247  NM_003057  NM_021977  NM_003054  NM_000240  NM_000898  NM_000524  NM_000863  NM_000864  NM_000865  NM_000866  NM_000621  NM_000867  NM_000868  NM_000869  NM_006028  NM_130770  NM_182537  NM_182589  NM_000870  NM_024012  NG_008325  NM_000871  NM_000872  NM_134442  NM_006272  NM_017521  NM_002509  NM_001174146  NM_032638  NM_001002295  NM_004316  NM_003924  NM_002144  NM_002145  NM_006168  NM_177400  NM_021728  NM_005269  NM_005270  NM_002007  NM_006119, NM_033165  NM_000193  NM_005631  NM_001485  NM_005258  NM_021977 | 107930  100650  182138  609149  602607  604842  193001  309850  309860  109760  182131  182133  182132  182134  182135  601122  312861  182139  604654  610121  610122  610123  602164  601305  -  601109  182137  123810  176990  607150  604612  602575  137295  131320  100790  603851  142968  142967  602563  605955  600037  165220  165230  164980  600483  600725  601500  601135  602437  604842 | Serotonin synthesis  Serotonin metabolism  Serotonin transport  Serotonin transport  Serotonin transport  Serotonin transport  Serotonin recycling  Serotonin receptor  Serotonin receptor  Serotonin receptor  Serotonin receptor  Serotonin receptor  Serotonin receptor  Serotonin receptor  Serotonin receptor  Serotonin receptor  Serotonin receptor  Serotonin receptor  Serotonin receptor  Serotonin receptor  Serotonin receptor  Serotonin receptor  Serotonin receptor  Serotonin receptor  Serotonin receptor  Serotonin receptor  Serotonin receptor  Transcription factor  Transcription factor  Transcription factor  Transcription factor  Transcription factor  Transcription factor  Transcription factor  Transcription factor  Transcription factor  Transcription factor  Transcription factor  Transcription factor  Transcription factor  Transcription factor  Transcription factor  Transcription factor  Transcription factor  Transcription factor  Transcription factor  Transcription factor  Transcription factor  Regulator  Serotonin transporter |  |

**Table S5: Candidate genes published to be associated with autism, autism spectrum disorders (ASDs) and ASD-related syndromes**

| **Protein name** | **Gene name** | **RefSeq IDs** | **OMIM Number** | **Association** | **Reference** |
| --- | --- | --- | --- | --- | --- |
| Aralkylamine N-acetyltransferase  Ataxin 2-binding protein  Adenosine deaminase  Adenylosuccinate lyase  Abelson helper intergration site 1  Aldolase A, fructose-biphosphate  Amyloid beta precursos protein-binding, family A, member 2  Apolipoprotein E  Androgen receptor  Aristaless related homeobox  Activating transcription factor 2  ATPase, class V, type 10A  Arginine vasopressin receptor 1A  Brain-derived neurotrophic factor  Calcium channel voltage-dependent L type alpha 1C subunit  Ca2+-dependent activator protein for secretion 2  Centaurin gamma 2  Cystic fribrosis transmembrane conductance regulator  Chimerin (chimaerin) 1  Contactin associated protein-like 2  Coatomer protein complex, subunit gamma 2  Coractin binding protein 2  Carboxypeptidase A1 (pancreatic)  Carboxypeptidase A5  Dopamine beta-hydroxylase  Doublecortin  **Aromatic L-amino acid decarboxylase**  7-dehydrocholesterol reductase  Disrupted in schizophrenia 1  Distal-less homeobox 1  Distal-less homeobox 2  Distal-less homeobox 6  Double C2-like Domain-containing protein, Alpha  Dopamine receptor D1  Dopamine receptor D2  Dopamine receptor D5  Engrailed homeobox 2  Fragil X retardation 1  Forkhead box P2  GABA A receptor, alpha 5  GABA A receptor, beta 3  GABA A receptor, gamma 3  Glutamate decarboxylase 1  Glycine receptor, alpha 2  Glutamate receptor ionotropic kainate 2  Glutamate receptor, metabotropic 3  Glutamate receptor, metabotropic 8  Gastrin-releasing peptide receptor  Hect domain and RLD 2  HIRA interacting protein 3  Homeobox A1  Homeobox B1  Homeobox D1  v-Ha-ras Harvey rat sarcoma viral oncogene homolog  **Serotonin receptor 2A**  **Serotonin receptor 7**  Inositol polyphosphate-1-phosphatase  Integrin, beta 3  Laminin, beta 1  Leucine rich repeat neuronal 3  **Monoamine oxidase A**  **Monoamine oxidase B**  MYC-associated zinc finger protein  Methyl CpG binding protein 2  Mesoderm specific transcript homolog  Met proto-oncogene  Melatonin receptor 1B  Neurobeachin  Necdin  Neurogenic differentiation 1  Neurofibromin 1  Neurofibromin 2  Neuroligin 3  Neuroligin 4, X-linked  Notch 4  Neuronal cell adhesion molecule  Neurexin1  Oxytocin receptor  Piccolo (presynaptic cytomatrix protein)  Proprotein convertase subtilisin/kexin type 2  Prodynorphin  Proenkephalin  Protein phosphatase 4, catalytic subunit  Phosphatase and tensin homologue  RAB3A, member RAS oncogene family  Reelin  Secretory carrier membrane protein 5  Sodium channel, voltage-gated, type I, alpha subunit  Sodium channel, voltage-gated, type II, alpha subunit  Sodium channel, voltage-gated, type III, alpha subunit  Secretin  Mitochondrial aspartate/glutamate carrier (AGC1)  Mitochondrial aspartate/glutamate carrier citrin  **Serotonin re-uptake transporter**  Solute carrier family 7, member 5  Seizure related 6 homolog (mouse)-like 2  Small nuclear ribonucleoprotein polypeptide N  SH3 and multiple ankyrin repeat domains 2  SH3 and multiple ankyrin repeat domains 3  Somatostatin receptor 5  Suppression of tumorigenicity 7  Syntaxin binding protein 6 (amisyn)  TAO kinase 2  T-box, brain, 1  Tryptophan 2,3-dioxygenase  Tyrosine Hydroxylase  Tuberous sclerosis 1  Tuberous sclerosis 2  Ubiquitin-conjugating enzyme E2H  Ubiquitin protein ligase E3A  Vasoactive intestinal peptide receptor 2  Tyrosine 3-monooxygenase/tryptophan 5-monooxygenase activation protein, zeta polypeptide  Wingless-type MMTV integration site family member 2 | *AANAT*  *A2BP1*  *ADA*  *ADSL*  *AHI1*  *ALDOA*  *APBA2*  *APOE*  *AR ARX*  *ATF2*  *ATP10C*  *AVPR1a*  *BDNF*  *CACNA1C*  *CADPS2*  *CENTG2*  *CFTR*  *CHN1*  *CNTNAP2*  *COPG2*  *CORTBP2*  *CPA1*  *CPA5*  *DBH*  *DCX*  ***DDC***  *DHCR7*  *DISC1*  *DLX1*  *DLX2*  *DLX6*  *DOC2A*  *DRD1*  *DRD2*  *DRD5*  *EN2*  *FMR1*  *FOXP2*  *GABRA5*  *GABRB3*  *GABRG3*  *GAD1*  *GLRA2*  *GRIK2*  *GRM3*  *GRM8*  *GRPR*  *HERC2*  *HIRIP3*  *HOXA1*  *HOXB1*  *HOXD1*  *HRAS1*  ***HTR2A***  ***HTR7***  *INPP1*  *ITGB3*  *LAMBI*  *LRRN3*  ***MAOA***  ***MAOB***  *MAZ*  *MECP2*  *MEST*  *MET*  *MTNR1B*  *NBEA*  *NDN*  *NEUROD1*  *NF1*  *NF2*  *NLGN3*  *NLGN4X*  *NOTCH4*  *NR-CAM*  *NRXN1*  *OXTR*  *PCLO*  *PCSK2*  *PDYN*  *PENK*  *PPP4C*  *PTEN*  *RAB3A*  *RELN*  *SCAMP5*  *SCN1A*  *SCN2A*  *SCN3A*  *SCT*  *SLC5A12*  *SLC13A13*  ***SLC6A4***  *SLC7A5*  *SEZ6L2*  *SNRPN*  *SHANK2*  *SHANK3*  *SSTR5*  *ST7*  *STXBP6*  *TAOK2*  *TBR1*  *TDO2*  *TH*  *TSC1*  *TSC2*  *UBE2H*  *UBE3A*  *VIPR2*  *YWHAZ*  *WNT2* | [NM_001088](http://www.ncbi.nlm.nih.gov/nuccore/NM_001088.2)  NM_018723  NM_000022  NM_000026  NM_017651  NM_000034  NM_005503  NM_000041  NM_000044  NM_139058  NM_001880  NM_024490  NM_000706  NM_170735  NM_000719  NM_017954  NM_001037131  NM_000492  NM_001822  NM_014141  NM_012133  NM_033427  NM_001868  NM_001127441  NM_000787  NM_178153  **NM_000790**  NM_001360  NM_018662  XM_087198  NM_004405  NM_005222  NM_003586  NM_000794  NM_000795  NM_000798  NM_001427  NM_002024  NM_014491  NM_000810  NM_000814  NM_033223  NM_000817  NM_001118885  NM_001166247  NM_000840  NM_000845  NM_005314  NM_004667  NM_003609  NM_005522  NM_002144  NM_024501  NM_176795  **­NM_000621**  **NM_000872**  NM_001128928  NM_000212  NM_002291  NM_018334  **NM_000240**  **NM_000898**  NM_002383  NM_004992  NM_002402  NM_000245  NM_005959  NM_015678  NM_002487  NM_002500  NM_000267  NM_000268  NM_018977  NM_020742  NM_004557  NM_001037132  NM_001135659  NM_000916  NM_014510  NM_002594  NM_001190892  NM_001135690  NM_002720  NM_000314  NM_002866  NM_005045  NM_138967 NM_006920  NM_021007  NM_006922  NM_021920  NM_003705  NM_014251  **NM_001045**  NM_003486.5  NM_012410  NM_003097  NM_012309  NM_001080420  NM_001053  NM_021908  NM_014178  NM_016151  NM_006593  NM_005651  NM_000360  NM_000368  NM_021055  NM_003344  NM_000462  NM_003382  NM_001135699  NM_003391 | 600950  605104  608958  608222  608894  103850  602712  107741  313700  300382  123811  605855  600821  113505  114205  609978  608651  602421  118423  604569  604355  609772  114850  609561  609312  300121  **107930**  602858  605210  600029  126255  600030  604567  126449  126450  126453  131310  309550  605317  137142  137192  600233  605363  305990  138244  601115  601116  305670  605837  603365  142955  142968  142987  190020  **182135**  **182137**  147263  173470  150240  -  **309850**  **309860**  600999  300005  601029  164860  600804  604889  602117  601724  613113  607379  300336  300427  164951  601581  600565  167055  604918  162151  131340  131330  602035  601728  179490  600514  613766  182389  182390  182391  82099  603667  603859  **182138**  600182  -  182279  603290  606230  182455  600833  607958  613199  604616  191070  191290  605284  191092  601082  601623  601970  601288  147870 | Autism  ASDs  Autism  Autism  ASDs  Autism  Autism  Autism  Autism  Autism  Autism  Autism  Autism/ASDs  Autism  ASDs/ASD-related syndromes  ASDs  ASD-related syndromes  Autism  Autism  ASDsASD-related syndromes  Autism  Autism  Autism  Autism  Autism  Autism  **Autism/Serotonin**  ASDs/ASD-related syndromes  ASDs  Autism  Autism  Autism  Autism  Autism  Autism  Autism  Autism/ASDs  Autism/ASDs/ASD-related syndromes  Autism  Autism  Autism/ASDs  Autism  Autism  Autism  Autism/ASDs  Autism  Autism  Autism  Autism  Autism  Autism  Autism  Autism  Autism  **Autism/Serotonin**  **Autism/Serotonin**  Autism  ASDs  Autism  Autism  **Autism/Serotonin**  **Autism/Serotonin**  Autism  Autism/ASDs/ASD-related syndromes  Autism  ASDs  Autism  Autism  Autism  Autism  Autism  Autism  Autism/ASDs  ASDs  Autism  Autism  ASDs  ASDs  Autism  Autism  Autism  Autism  Autism  ASDs  Autism  Autism/ASDs  Autism  Autism  Autism  Autism  Autism  Autism/ASDs  Autism  **Autism/ASDs/Serotonin**  Autism  Autism  Autism  ASDs/ASD-related syndromes  ASDs/ASD-related syndromes  Autism  Autism  Autism  Autism  Autism  Autism  Autism  Autism/ASDs/ASD-related syndromes  Autism/ASDs/ASD-related syndromes  Autism  Autism/ASDs/ASD-related syndromes  Autism  Autism  Autism |  |

* In bold are the candidate genes which have been published to be involved both in serotonin and autism/ASDs/ASD-related syndromes.

References

1. Haavik J, Blau N, Thony B (2008) Mutations in human monoamine-related neurotransmitter pathway genes. Hum Mutat 29: 891-902.

2. Keung WM, Vallee BL (1998) Daidzin and its antidipsotropic analogs inhibit serotonin and dopamine metabolism in isolated mitochondria. Proc Natl Acad Sci U S A 95: 2198-2203.

3. Ichikawa M, Okamura-Oho Y, Okunishi R, Kanamori M, Suzuki H, et al. (2005) Expression analysis of genes responsible for serotonin signaling in the brain. Neurobiol Dis 19: 378-385.

4. Engel K, Zhou M, Wang J (2004) Identification and characterization of a novel monoamine transporter in the human brain. J Biol Chem 279: 50042-50049.

5. Schmitt A, Mossner R, Gossmann A, Fischer IG, Gorboulev V, et al. (2003) Organic cation transporter capable of transporting serotonin is up-regulated in serotonin transporter-deficient mice. J Neurosci Res 71: 701-709.

6. Scott MM, Deneris ES (2005) Making and breaking serotonin neurons and autism. Int J Dev Neurosci 23: 277-285.

7. Pattyn A, Simplicio N, van Doorninck JH, Goridis C, Guillemot F, et al. (2004) Ascl1/Mash1 is required for the development of central serotonergic neurons. Nat Neurosci 7: 589-595.

8. Alenina N, Bashammakh S, Bader M (2006) Specification and differentiation of serotonergic neurons. Stem Cell Rev 2: 5-10.

9. Thöny B, Blau N (2006) Mutations in the BH4-metabolizing genes GTP cyclohydrolase I, 6-pyruvoyl-tetrahydropterin synthase, sepiapterin reductase, carbinolamine-4a-dehydratase, and dihydropteridine reductase. Hum Mutat 27: 870-878.

10. Daws LC (2009) Unfaithful neurotransmitter transporters: focus on serotonin uptake and implications for antidepressant efficacy. Pharmacol Ther 121: 89-99.

11. Anderson BM, Schnetz-Boutaud NC, Bartlett J, Wotawa AM, Wright HH, et al. (2009) Examination of association of genes in the serotonin system to autism. Neurogenetics 10: 209-216.

12. Abrahams BS, Geschwind DH (2008) Advances in autism genetics: on the threshold of a new neurobiology. Nat Rev Genet 9: 341-355.

13. Wassink TH, Brzustowicz LM, Bartlett CW, Szatmari P (2004) The search for autism disease genes. Ment Retard Dev Disabil Res Rev 10: 272-283.

14. Kumar RA, Marshall CR, Badner JA, Babatz TD, Mukamel Z, et al. (2009) Association and mutation analyses of 16p11.2 autism candidate genes. PLoS One 4: e4582.

15. Andres C (2002) Molecular genetics and animal models in autistic disorder. Brain Res Bull 57: 109-119.

16. Castermans D, Wilquet V, Steyaert J, Van de Ven W, Fryns JP, et al. (2004) Chromosomal anomalies in individuals with autism: a strategy towards the identification of genes involved in autism. Autism 8: 141-161.

17. Castermans D, Volders K, Crepel A, Backx L, De Vos R, et al. (2010) SCAMP5, NBEA and AMISYN: three candidate genes for autism involved in secretion of large dense-core vesicles. Hum Mol Genet 19: 1368-1378.

18. Ramoz N, Reichert JG, Smith CJ, Silverman JM, Bespalova IN, et al. (2004) Linkage and association of the mitochondrial aspartate/glutamate carrier SLC25A12 gene with autism. Am J Psychiatry 161: 662-669.

19. Berkel S, Marshall CR, Weiss B, Howe J, Roeth R, et al. (2010) Mutations in the SHANK2 synaptic scaffolding gene in autism spectrum disorder and mental retardation. Nat Genet 42: 489-491.
